# The Dose-Dependent Influence of Type 2 Resistant Starch on Gut Microbial Communities and Metabolic Outputs: An In Vitro Simulation

**DOI:** 10.3390/foods14183255

**Published:** 2025-09-19

**Authors:** Huowang Zheng, Fangshu Shi, Jinjun Li, Xiangyu Bian, Shuisheng Wu, Xiaoqiong Li

**Affiliations:** 1 College of Pharmacy, Fujian University of Traditional Chinese Medicine, Fuzhou 350122, China; 17850859173@139.com; 2 State Key Laboratory for Quality and Safety of Agro-Products, Institute of Food Sciences, Zhejiang Academy of Agricultural Sciences, Hangzhou 310021, China; shifangshu1992@163.com (F.S.); lijinjun@zaas.ac.cn (J.L.); bianjinfeng1993@163.com (X.B.)

**Keywords:** in vitro fermentation, type 2 resistant starch, gut microbiota, tryptophan metabolism

## Abstract

This study systematically investigated the dose–response relationship of resistant starch type 2 (RS2; Hi-maize 260; 0–15 g/L) on gut microbial composition, short-chain fatty acid (SCFA)/gas output, and tryptophan catabolism using an in vitro fermentation model. The highest RS2 concentration (15 g/L) elicited optimal metabolic outcomes, including maximal SCFA production; significant H_2_S reduction; and redirected tryptophan metabolism from potentially detrimental indoles toward neuroprotective metabolites. Microbial profiling revealed dose-dependent enrichment of saccharolytic taxa (*Bifidobacterium*, *Lactobacillus*) with concomitant suppression of proteolytic pathobionts (e.g., *Escherichia-Shigella*). Correlation analyses revealed strong positive associations between beneficial microbes and both SCFAs and neuroprotective metabolites, whereas pathogenic taxa correlated inversely with these compounds. Collectively, these findings establish that functionally relevant microbiome modulation requires a sufficiently high, dose-tailored intake of RS2, providing a rational basis for precision dietary strategies aimed at improving host metabolic and gut health.

## 1. Introduction

Type 2 resistant starch (RS2), classified as a fermentable dietary fiber, resists digestion by human amylases in the small intestine but undergoes microbial fermentation in the colon [1,2]. Accumulating evidence indicates that RS2 confers multiple physiological benefits, including modulation of the gut microbiome, prevention of colitis, anti-inflammatory effects, and improved glucose homeostasis [3,4,5,6,7]. The primary mechanism underlying these benefits is the microbial fermentation of RS2 into short-chain fatty acids (SCFAs)—particularly acetate, propionate, and butyrate [8]. These SCFAs contribute to colonic health by promoting beneficial bacteria such as bifidobacteria and maintaining epithelial integrity [9]. Notably, RS2 serves as a preferential substrate for butyrogenic bacteria, including *Eubacterium rectale* and *Bifidobacterium* species [10]. The resultant butyrate is crucial for maintaining gastrointestinal integrity and may contribute to systemic metabolic regulation, particularly in glucose and lipid homeostasis [11,12]. Despite these established benefits, the dose-dependent effects of RS2 on microbial composition modulation and activities remain insufficiently characterized.

Although current dietary guidelines recommend a daily fiber intake of 25–30 g for adults [13], numerous animal studies employ supraphysiological RS2 concentrations (e.g., 20–40% of diet) to demonstrate benefits such as reduced DNA damage and enhanced butyrogenesis [14,15,16]. While these studies confirm the efficacy of RS2 in improving weight management and insulin sensitivity, the translational relevance of these elevated doses to human nutrition remains unclear. Human trials indicate that daily RS2 supplementation at 30–60 g improves insulin sensitivity and glycaemic control [17,18,19], with meta-analyses further corroborating that interventions lasting ≥ 8 weeks with doses ≥ 26 g/day are most effective—especially among overweight or prediabetic individuals [20]. This dose disparity complicates the extrapolation of mechanistic insights from animal models to human physiology. Moreover, the specific dose-dependent influences of RS2 on microbial ecology and functional outputs have not been thoroughly characterized at physiologically relevant concentrations. Although cumulative evidence supports a dose–response relationship between RS2 intake and metabolic improvement, the mechanistic basis underlying this association has not been fully elucidated.

In vitro fermentation models provide a valuable platform for bridging this gap, enabling precise control over substrate dosage within a physiologically relevant range under tightly controlled conditions. These systems allow direct examination of microbial community dynamics and metabolic outputs without interference from host physiology [21]. Tryptophan (Trp), an essential aromatic amino acid, serves as a precursor for numerous bioactive metabolites that play critical roles in immune and neurological regulation, primarily via the kynurenine and serotonin pathways [22]. In addition, the gut microbiota actively participates in Trp metabolism through the production of various catalytic enzymes that sequentially degrade Trp into indole and its derivatives. These microbial metabolic products significantly influence host physiology, participating in processes ranging from plasma protein turnover to the biosynthesis of essential molecules including niacin and melatonin [23]. A recent study has revealed that carbohydrate availability directs microbial tryptophan metabolism through metabolic interactions within the gut community, and that dietary fibre supplementation can inhibit indole production in complex microbial ecosystems [24].

Given that RS2 fermentation profoundly alters gut microbial structure—shifting the balance from proteolytic to saccharolytic metabolism—it is likely to substantially modulate the fate of dietary tryptophan. Despite growing recognition of the gut microbiota’s role in Trp metabolism, the dose-dependent effects of RS2 on tryptophan metabolic pathways remain poorly characterized, especially in well-controlled in vitro systems.

Here, we employed a 48 h in vitro fermentation model to characterize the dose–response effects of RS2 (0–15 g L^−1^, encompassing physiologically relevant human intake levels) on microbial composition, SCFA and gas profiles, and tryptophan catabolism. We hypothesized that RS2 at higher doses would promote a metabolic profile associated with enhanced gut and systemic health, with 15 g L^−1^ representing an effective threshold for meaningful modulation of the gut microbiome. These findings offer a rational basis for developing precision dietary interventions using appropriately dosed RS2 formulations.

## 2. Materials and Methods

### 2.1. Materials and Reagents

High-amylose cornstarch (Hi-maize 260, RS2, 66.4% dietary fiber) was purchased from Ingredion Food Ingredients Co., Ltd. (Shanghai, China). Tryptophan, 2-Oxindole, indole-3-lactic acid and indole-3-propinic standard were supplied by Aladdin Biochemical (Shanghai, China) Technology Co., Ltd. Indole-3-aldehyde and tryptamine standard were purchased from Yuanye Bio-Technology Co., Ltd. (Shanghai, China). Kynurenine, indole, 3-indoleacrylicacid, indole-3-acetic acid and 5-hydroxytryptamine were obtained from Sigma-Aldrich (Shanghai, China). All other reagents used were of analytical purity.

### 2.2. In Vitro Fermentation

The method of in vitro fermentation referred to the pre-experimental protocols [25]. The fermentation medium (1.0 L) was composed of NaCl (0.9 g), K_2_HPO_4_ (0.45 g), KH_2_PO_4_ (0.45 g), CaCl_2_·6H_2_O (0.009 g), MgSO_4_·7H_2_O (0.09 g), tryptone (10 g), yeast extract (2.5 g), L-cysteine (1 g), vitamin H (0.0005 g), vitamin B-12 (0.0005 g), vitamin B-6 (0.0075 g), p-aminobenzoic acid (0.0015 g) and folic acid (0.0025 g) and 1 mL of resazurin was added to the medium as a redox indicator. After preparation, the medium was sterilized at 121 °C for 30 min. After cooling, vitamins were added as a filter-sterilized solution to the medium. This precaution was taken to prevent the thermal degradation and denaturation of heat-sensitive vitamins during the autoclaving process, thereby ensuring their bioavailability throughout the fermentation experiment.

Fecal specimens were obtained from eight healthy adult volunteers (gender-balanced cohort: 4 males, 4 females; age range: 22–35 years; BMI: 18.5–23.9 kg/m^2^) residing in Hangzhou, China. All participants met stringent inclusion criteria: (1) no history of gastrointestinal disorders (including inflammatory bowel disease, irritable bowel syndrome, or colorectal cancer); (2) absence of antibiotic, probiotic, or prebiotic use within the preceding three months; and (3) maintenance of normal dietary habits during the study period. Complete demographic characteristics and baseline health parameters are provided in Supplementary Table S1. Samples were collected into centrifuge tubes filled with sterile PBS and diluted to 0.1 g·mL^−1^. The fecal suspension was filtered with fecal bacteria filter, and the liquid on the clarified side was absorbed for use. Before the study commenced, the written informed consent was acquired from all volunteers and the experimental was approved by the Ethics Committee of Zhejiang Academy of Agricultural Sciences (No. 2023–025).

To simulate the fermentation process of RS2 in the human gut, a 10 mL anaerobic fermentation vial was used as a reaction site for in vitro fermentation simulation. To initiate fermentation, each vial received 5 mL of sterile nutritive medium (with or without RS_2_), 0.5 mL tryptophan solution (5 mM) and 0.5 mL faecal inoculum (final 5% *v*/*v*). RS2 was added at 5, 10 or 15 g L^−1^, corresponding to human-equivalent daily intakes of 3.75, 7.5 and 11.25 g for an average colonic volume of 0.75 L—amounts readily achievable through diets rich in legumes, whole grains or cooked-then-cooled starchy foods [26]. These concentrations defined the low- (LRS2), medium- (MRS2) and high-dose (HRS_2_) groups, which were compared with a negative control lacking RS2 (NC).

Simulated in vitro fermentation experiments were performed in a thermostatic incubator (37 °C). Samples were collected at 0, 6, 12, 24, and 48 h, respectively, and were split, labeled, and stored at −80 °C for subsequent analysis. All experiments were performed in triplicate.

### 2.3. PH Determination

During the fermentation process, 0.5 mL of sample was collected at 0 h, 6 h, 12 h, 24 h and 48 h using a sterile 1 mL disposable syringe, and tested using a pen pH meter.

### 2.4. Determination of Short-Chain Fatty Acids (SCFAs) by GC

Gas chromatography (GC2010plus, Shimadzu Corporation, Kyoto, Japan) was used to quantify SCFA concentrations (acetic, propionic, isobutyric, butyric, isovaleric, and valeric acids) according to established methods [27] with modifications. Briefly, fermentation samples were acidified with crotonic metaphosphate solution, centrifuged, and filtered. The supernatant was injected into a gas chromatograph equipped with a flame ionization detector (FID) and a DB-FFAP capillary column. Detailed chromatographic parameters (e.g., temperature program, flow rates) are provided in Supplementary Table S2.

### 2.5. Testing In Vitro Fermentation Gas Production

After 48 h of fermentation, the samples were removed for gas detection. Specifically, gas analysis was carried out using an FGA-050 intestinal microbial fermentation gas analyzer (Hangzhou Hailu Biological Company). The analyzer was calibrated daily using certified standard gas mixtures (Hailu Biological, Hangzhou, China). The first step is to clean the gas analyzer system, open the detection interface and the valve of the gas inlet valve. After cleaning, the sample was placed into the instrument, and the concentrations of the gas components—carbon dioxide (CO_2_), methane (CH_4_), hydrogen (H_2_), and hydrogen sulfide (H_2_S)—were measured [28]. The limits of detection (LOD) were 5 ppm.

### 2.6. Detecting In Vitro Fermentation Tryptophan Metabolites

According to preliminary experiments [29,30], the methanol (800 µL) was added to 200 µL of fermentation broth, vortexed, and ultrasonicated for 5 min to accelerate extraction. Then, it was placed in the refrigerator (−20 °C) for 10 min to accelerate precipitation. The sample was then centrifuged at 14,000 rpm (4 °C, 10 min). Liquid chromatography/tandem mass spectrometry (LC/MS-MS) analysis was used to analyse tryptophan metabolite levels in samples. The metabolite included 2-Oxindole (2-OX), indole-3-propinic (IPA), indole-3-acetic acid (IAA), kynurenine (KYN), Indole, indole-3-lactic acid, Tryptophan, indole-3-aldehyde (IALD), Tryptamine (TA) and 5-hydroxytryptamine (5-HT) in fermentation broth. The LC-MS/MS system comprised a QTRAP™ 6500 mass spectrometer (Sciex, Framingham, MA, USA) coupled with an AQUITY UPLC^®^ system (I-class, Waters, MA, USA), featuring a temperature-controlled autosampler and a UPLC binary pump (I-class, Waters). Separation was performed on an ACQUITY PREMIER BEH C18 column (1.7 μm, 2.1 × 150 mm; Waters) maintained at 45 °C. System operation and data acquisition were managed using Analyst^®^ software (v1.6.2, Sciex) [25].

### 2.7. DNA Extraction and 16S rRNA Gene Sequencing

First, 1.6 mL of the sample was centrifuged after 48 h of fermentation for 10 min (8000× *g*, 4 °C). The supernatant was removed, and the precipitated sample was sent to Minco Biotech in Hangzhou, China, for 16S rRNA gene sequencing. The FastLee DNA kit for Feces (Hangzhou Legenomics Bio-Pharm Technology Co., Ltd., Hangzhou, China) was used to extract total genome DNA from samples. DNA concentration and purity were monitored on 1% agarosegels. And we used sterile water to dilute the DNA to 1 ng/μL according to the concentration of the assay. Then, we amplified the 16S rRNA gene using the universal primers 341F (5′-CCTAYGGGRBGCASCAG-3′) and 806R (5′-GGACTACNNGGGTATCTAAT-3′). Each 30 μL PCR reaction contained 15 μL Phusion^®^ High-Fidelity PCR Master Mix (New England Biolabs), 0.2 μM of each primer, and approximately 10 ng of genomic DNA templates. Thermal cycling conditions comprised initial denaturation (98 °C, 1 min); 30 cycles of denaturation (98 °C, 10 s), annealing (50 °C, 30 s), and extension (72 °C, 60 s); followed by a final extension (72 °C, 5 min). PCR amplicons were subsequently purified and assessed for quality and concentration [31]. At first, the same volume of 1 × loading buffer (contained SYBR green) was mixed with PCR products. Then, we performed electrophoresis on 2% agarose gel for detection. The samples exhibiting bright main bands and the band sizes ranging from 400 to 450 base pairs (bp) were selected for further experiments. The subsequent steps involved the mixing and purification of the PCR products. PCR products were mixed in equidensity ratios. Finally, to purify the PCR product mixture, we used the GeneJET Gel Extraction Kit (Thermo Scientific, Waltham, MA, USA).

Finally, DNA sequencing libraries were prepared with the NEBNext^®^ Ultra™ DNA Library Prep Kit for Illumina (New England Biolabs (NEB), Ipswich, MA, USA) according to the manufacturer’s protocol, with unique index barcodes incorporated for sample multiplexing. Library quality control was performed using a Qubit^®^ 2.0 Fluorometer (Thermo Fisher Scientific) for quantification and an Agilent Bioanalyzer 2100 for size distribution analysis. Finally, paired-end sequencing (250/300 bp read length) was conducted on an Illumina MiSeq platform [32]. The 16S rRNA gene sequencing data are available in the NCBI Sequence Read Archive database under accession number (PRJNA1199588).

Sequences analysis was performed by the UPARSE software package using the UPARSE-OTU and UPARSE-OTUref algorithms. In-house Perl scripts were used to analyze alpha (ACE, Chao, Shannon, Simpson) and beta (Principal Coordinate Analysis) diversity. We used Bray–Curtis distance for Principal Coordinate Analysis (PCoA). Metastats software (version 3.3.1) analysis was used to confirm whether there were differences in individual taxonomic abundance between the two groups and to quantify biomarkers within the different groups using Linear Discriminant Analysis (LDA) Effect Size (LEfSe) analysis. Additionally, Spearman correlations were used to associate differential taxa with SCFAs, gas production, and tryptophan metabolites [33]. We used Wilcoxon rank-sum test and Welch’s *t*-test to compare the data, and *p* < 0.05 was considered a significant difference between the two sets of data.

### 2.8. Statistical Analysis

Graphs were plotted using GraphPadPrism 9.5 and data were processed and analyzed using SPSS 24, with data results displayed as mean ± standard error. Data were analyzed for differences using one-way analysis of variance (ANOVA) combined with Tukey’s post hoc test, setting *p* < 0.05 as statistically significant.

## 3. Results

### 3.1. RS2 Effects on Microbial Communities

#### 3.1.1. RS2 Effects on Microbial Diversity

**Figure 1:**
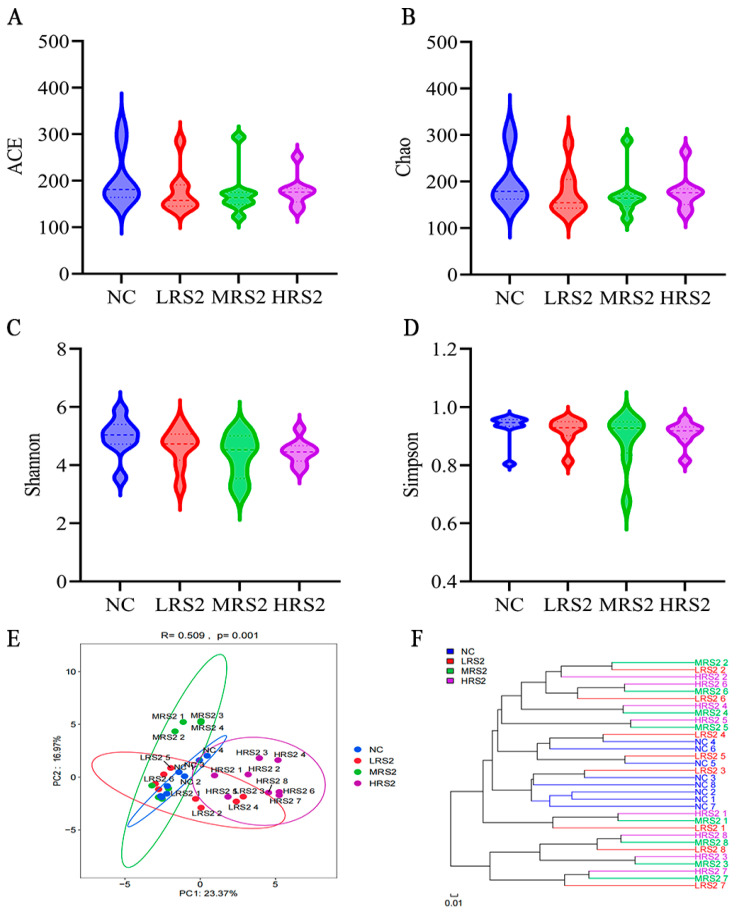
Effect of graded concentrations of RS2 on alpha- and beta-diversity after 48 h of in vitro fermentation. (**A**): ACE index, (**B**): Chao1 index, (**C**): Shannon index, (**D**): Simpson index, (**E**): principal-coordinate analysis (PCoA) and (**F**): Hierarchical clustering. Experimental treatments included NC (negative control, 0 g/L), LRS2 (5 g/L), MRS2 (10 g/L), and HRS2 (15 g/L) of Hi-Maize 260 (*n* = 8 per group, with 4 female and 4 male replicates). One-way ANOVA with Tukey’s post hoc test was performed.

Gut microbiota plays a crucial role in the fermentation process. The α-diversity can reflect the evenness and richness of microbial communities [34]. None of the α-diversity indices (ACE, Chao1, Shannon, or Simpson) showed statistically significant differences among the four groups (*p* > 0.05, Figure 1A–D). Although numerical trends were observed—such as increases in ACE and Chao1 (richness indice) with higher RS2 doses (albeit remaining below NC levels) and a slight decrease in Shannon index (community diversity indice) with increasing RS2 concentration—these patterns were not statistically supported. This pattern of reduced overall richness was further corroborated by the Venn diagram (Supplementary Figure S1), which showed a decrease in the number of unique operational taxonomic units (OTUs) in all RS2-treated groups compared to the NC group.

Moreover, β-diversity reflects the differences in microbial community composition and distribution between samples. The distance between the NC, LRS2 and MRS2 groups was observed to be relatively close (R = 0.5091, *p* < 0.01; Figure 1E), which may indicate that these groups exhibited somewhat similar regulatory effects on microorganisms. In contrast, the distance between the HRS2 group and the other groups was comparatively far, potentially suggesting that the higher concentration of RS2 a more distinct microbial community structure. As shown in Figure 1F, hierarchical clustering based on Bray–Curtis distance showed that the LRS2 group was clustered more closely with the NC group, while the MRS2 and HRS2 groups formed separate clusters. Specifically, the clustering analysis indicated that the species composition of the NC and LRS2 groups was more similar, whereas the NC group was more distinct from the MRS2 and HRS2 groups. These patterns could imply that low-dose RS2 treatment had a less pronounced impact on microbial composition compared to medium and high doses.

#### 3.1.2. RS2 Effects on Microbial Components

**Figure 2:**
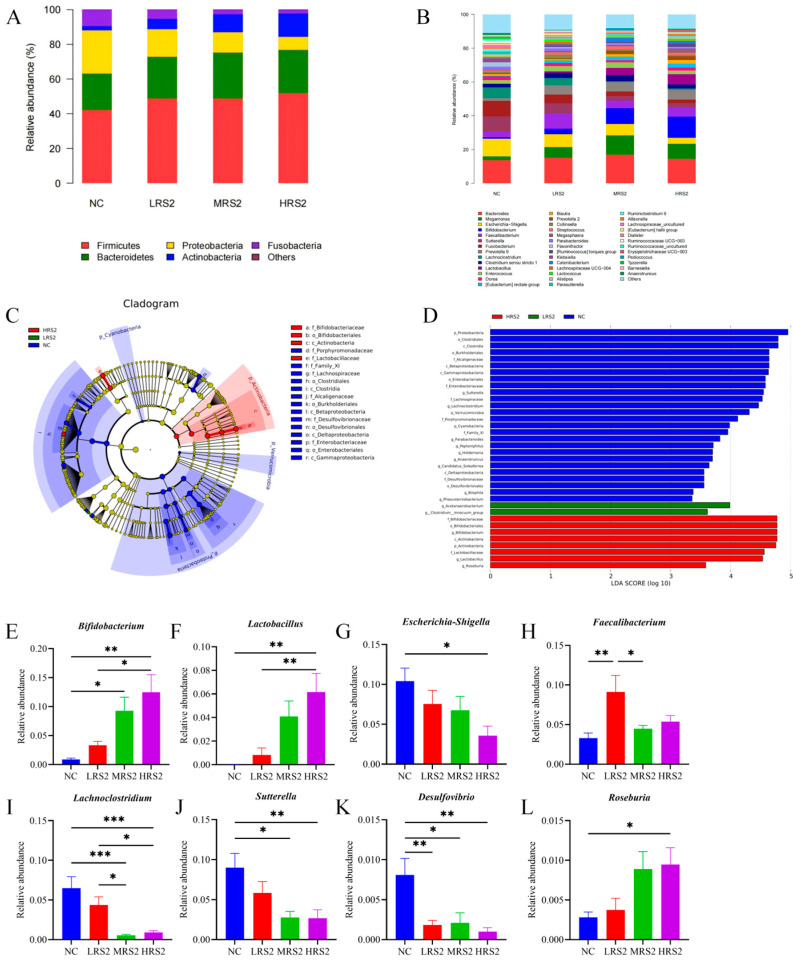
Relative abundance of bacteria at (**A**): the phylum level, (**B**): genus level, (**C**): LDA score and (**D**): Lefse analysis of gut microbiota. Relative abundance of significantly different bacterial genera at genus level ((**E**): *Bifidobacterium*, (**F**): *Lactobacillus*, (**G**): *Escherichia-Shigella*, (**H**): *Faecalibacterium*, (**I**): *Lachnoclostridium*, (**J**): *Sutterella*, (**K**): *Desulfovibrio* and (**L**): *Roseburia*)). Experimental treatments included NC (negative control, 0 g/L), LRS2 (5 g/L), MRS2 (10 g/L), and HRS2 (15 g/L) of Hi-Maize 260 (*n* = 8 per group, with 4 female and 4 male replicates). One-way ANOVA with Tukey’s post hoc test was performed. Significant correlations were marked by * *p* < 0.05; ** *p* < 0.01; *** *p* < 0.001.

At the phylum level (Figure 2A), the microbial community after fermentation was mainly composed of *Firmicutes*, *Bacteroidetes*, *Proteobacteria* and *Actinobacteria*. Compared with the NC group, the addition of RS2 increased the abundance of *Firmicutes*, *Actinobacteria*, and *Bacteroides*, while decreasing the abundance of *Proteobacteria* and *Fusobacteria*. Additionally, at the genus level (Figure 2B), RS2 increased the abundance of *Bifidobacterium*, *Prevotella 9*, *Megamonas* and *Lactobacillus* compared to the NC group. Conversely, the abundance of *Escherichia-Shigella*, *Lachnoclostridium*, *Fusobacterium* and *Sutterella* was decreased. Thus, the changes in the gut microbiota induced by RS2 exhibited a dose-dependent gradient effect.

LEfSe was used to identify differential bacteria groups among various samples. LDA score was used to analyze differences in gut microbiota under different sample interventions, with higher LDA score indicating more significant difference. Different doses of RS2 exhibited distinct effects on bacterial enrichment. As could be seen in Figure 2C,D, specifically, the LRS2 group promoted the growth of *Cetanaerobacterium*, *Anaerovoracaceae*, and *Clostridium__innocuum*_group. In contrast, the HRS2 group significantly increased the abundance of *Bifidobacteriaceae*, *Bifidobacteriales*, *Bifidobacterium*, and *Actinobacteria*. Notably, the MRS2 group had no enrichment effect on bacteria.

Figure 2E–L displayed eight bacterial taxa at the family/genus level with significant differences in one-way ANOVA tests among groups. The results demonstrated that compared to the NC group, the HRS2 group exhibited significantly higher relative abundances of *Bifidobacterium*, *Lactobacillus*, and *Roseburia*, while the relative abundances of *Escherichia-Shigella*, *Lachnoclostridium*, *Sutterella*, and *Desulfovibrio* were significantly lower.

### 3.2. Changes in PH and SCFA Compositions During RS2 Fermentation

**Figure 3:**
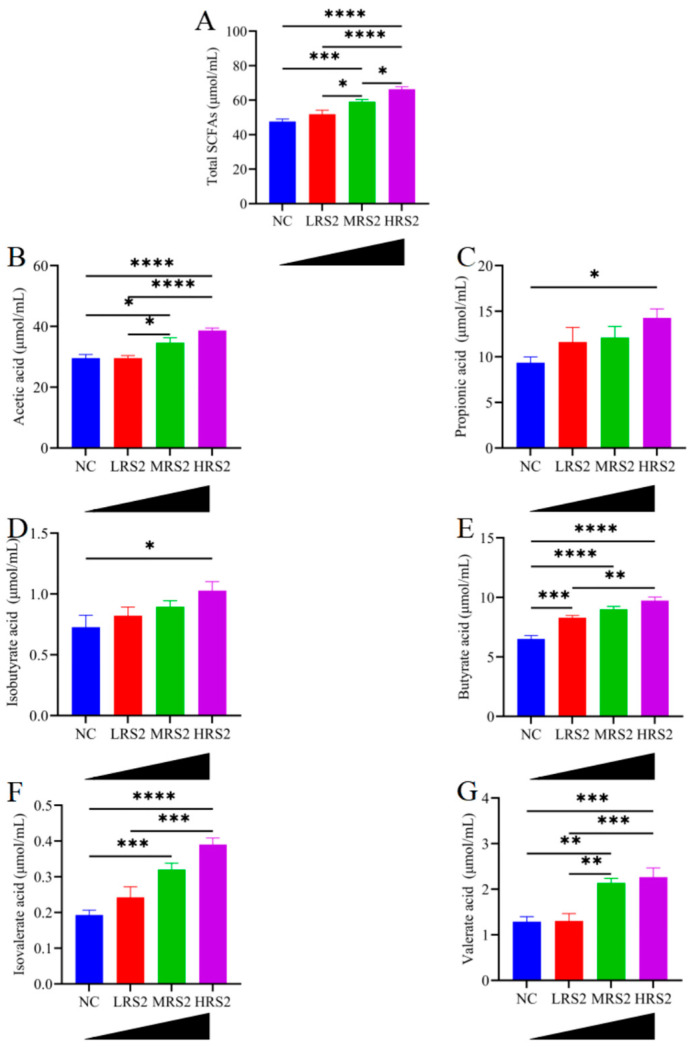
Changes in SCFA during fermentation. (**A**): Total SCFAs, (**B**): acetic acid, (**C**): propionic acid, (**D**): isobutyric acid, (**E**): butyric acid, (**F**): isovaleric acid and (**G**): valeric acid. Experimental treatments included NC (negative control, 0 g/L), LRS2 (5 g/L), MRS2 (10 g/L), and HRS2 (15 g/L) of Hi-Maize 260 (*n* = 8 per group, with 4 female and 4 male replicates). One-way ANOVA with Tukey’s post hoc test was performed. Significant correlations were marked by * *p* < 0.05; ** *p* < 0.01; *** *p* < 0.001, **** *p* < 0.0001.

SCFAs, which are fatty acids with 1–6 carbon atoms, are also byproducts of intestinal microbiota fermenting undigested dietary fiber in the colon [35]. We tested the fecal SCFA content in RS2-treated groups by gas chromatography (GC). Specifically, after 48 h of fermentation, the contents of acetic acid (Figure 3B), valeric acid (Figure 3G) and isovaleric acid (Figure 3F) in MRS2 and HRS2 groups were significantly higher than those in the NC group. The detailed time-course profiles (0–48 h) for these acids are provided in Supplementary Figure S2.

The HRS2 treatment group showed significantly higher levels of propionic acid (Figure 3C), butyric acid (Figure 3E), and isobutyric acid (Figure 3D), compared to the NC group. Additionally, the total SCFA output rose progressively from LRS2 to HRS2, reaching its apex at 15 g/L. These results indicated that SCFA production increased with elevating RS2 dosage, although significant increases in specific fatty acids such as propionic acid and isobutyric acid were only observed at a sufficiently high concentration (15 g/L). The detailed time-course profiles (0–48 h) for these acids are provided in Supplementary Figure S2.

### 3.3. RS2 Dose-Dependently Increased Total Gas Production

**Figure 4:**
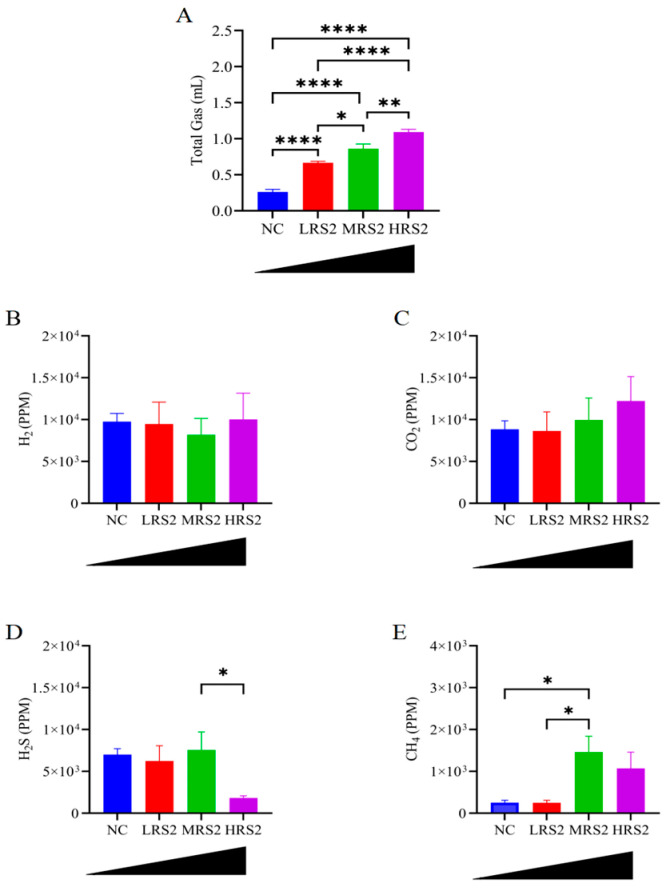
Gas production during in vitro fermentation of RS2 by human gut microbiota. (**A**): Total gas production, (**B**): hydrogen (H_2_), (**C**): carbon dioxide (CO_2_), (**D**): hydrogen sulfide (H_2_S), and (**E**): methane (CH_4_). Experimental treatments included: NC (negative control, 0 g/L), LRS2 (5 g/L), MRS2 (10 g/L), and HRS2 (15 g/L) of Hi-Maize 260 (*n* = 8 per group, with 4 female and 4 male replicates). One-way ANOVA with Tukey’s post hoc test was performed. Significant correlations were marked by * *p* < 0.05; ** *p* < 0.01; **** *p* < 0.0001. Absence of symbols denotes no significant difference among the four groups.

RS2 usually produces gas during fermentation by gut microbiota, and these gases can affect the dilation of the colon wall, which in turn has an impact on the rate of passage of substances through the colon. Therefore, gas production is considered as one of the important indicators of the prebiotic potential and microbial utilization of RS2 [36]. Gas production can also be used as an index to evaluate the extent of microbial RS2 utilization. As shown in Figure 4A, total gas production increased significantly after adding RS2 compared with the NC group, exhibiting a dose-dependent effect. Specifically, while H_2_ (Figure 4B) and CO_2_ (Figure 4C) content did not differ significantly among groups, the HRS2 group had the highest gas production. Notably, H_2_S content was similar among the NC, LRS2 and MRS2 groups, but the HRS2 group exhibited a significant decrease compared to the MRS2 group (Figure 4D). For CH_4_, the MRS2 group had a significantly higher level than the NC group, and the HRS2 also showed an increasing trend (Figure 4E). In conclusion, the microbial community in the HRS2 group demonstrated the highest utilization degree of RS2.

### 3.4. Effect of RS2 on Tryptophan Metabolites After 48 h Fermentation

**Figure 5:**
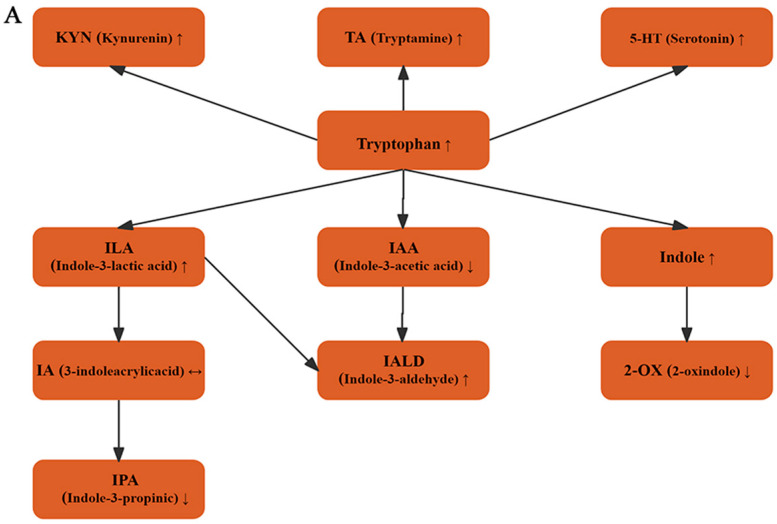

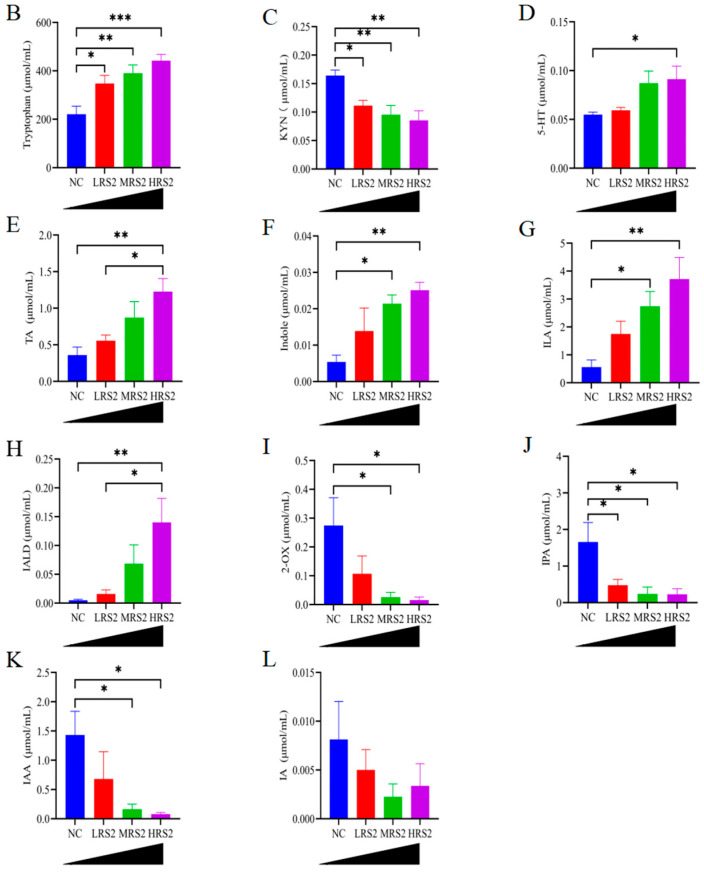
RS2 affects the tryptophan metabolites produced by human gut microbiota during the fermentation. (**A**): Tryptophan metabolism pathway diagram, (**B**): Tryptophan, (**C**): KYN, (**D**): 5-HT, (**E**): TA, (**F**): Indole, (**G**): ILA, (**H**): IALD, (**I**): 2-OX, (**J**): IPA, (**K**): IAA and (**L**): IA. Experimental treatments included NC (negative control, 0 g/L), LRS2 (5 g/L), MRS2 (10 g/L), and HRS2 (15 g/L) of Hi-Maize 260 (*n* = 8 per group, with 4 female and 4 male replicates). One-way ANOVA with Tukey’s post hoc test was performed. Significant correlations were marked by * *p* < 0.05; ** *p* < 0.01; *** *p* < 0.001. Absence of symbols denotes no significant difference among the four groups. Upward arrows represent an increase in content, while downward arrows represent a decrease.

Tryptophan serves as a vital substrate for microbial metabolism within the gut, exerting significant influence on gut health, immune function, and overall host metabolism [37]. The concentration of tryptophan was significantly higher in all RS2-treated groups than in the NC group (Figure 5B). Notably, compared with the NC group, the concentration of KYN in the kynurenine metabolic pathway significantly decreased as the concentration of RS2 fermentation supernatant increased (Figure 5C). This indicates that RS2 fermentation may suppress the activity of the kynurenine metabolic pathway. Within the 5-HT pathway, the concentrations of TA (Figure 5D) and 5-HT (Figure 5E) in the HRS2 group were significantly higher than the NC group, which suggests that RS2 fermentation may enhance the activity of the 5-HT metabolic pathway, potentially promoting the synthesis and release of 5-HT [38]. In addition, the concentrations of indole (Figure 5F), indole-3-lactic acid (ILA) (Figure 5G), and IALD (Figure 5H) were elevated in the RS2 treatment group, whereas the concentrations of 2-OX (Figure 5I), IPA (Figure 5J), and IAA (Figure 5K) were reduced compared with the NC group in the indole pathway. Notably, 3-indoleacrylic acid (IA) was unaffected by RS2 treatment (Figure 5L). The increase in concentration may be attributed to high-dose RS2 acting as a carbon source to stimulate the proliferation of lactic acid bacteria, thereby enhancing their tryptophan degradation capacity and generating more indole metabolites. Conversely, the decrease likely results from HRS2 favoring microbial utilization of the ILA pathway (Figure 5A) while suppressing alternative pathways, such as the production of IPA and IAA [39,40]. These findings indicate that RS2 fermentation modulates the indole metabolic pathway and promotes the production of beneficial metabolites such as ILA.

### 3.5. Correlation Analysis of SCFAs, Gas and Tryptophan Metabolites with Bacteria

**Figure 6:**
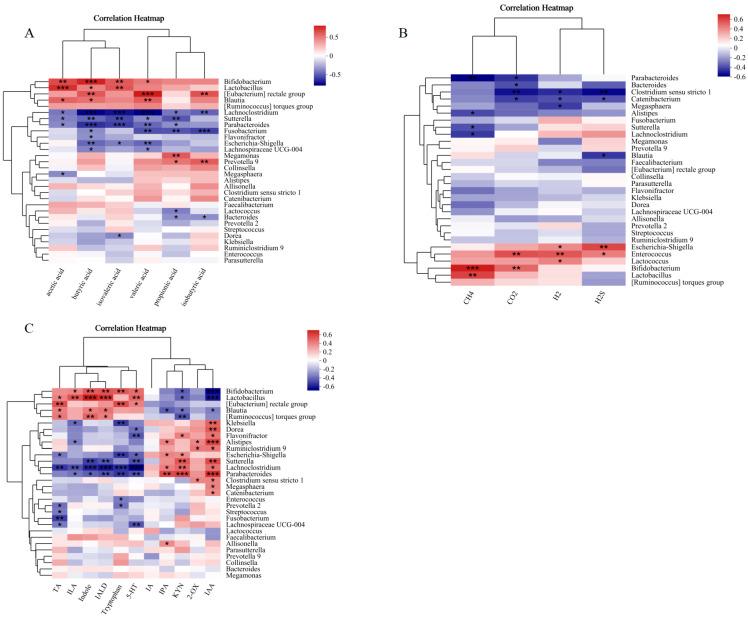
Correlations between SCFAs and tryptophan metabolites with top 30 abundance of gut microbiota during the RS2 fermentation. Spearman correlation heatmap of (**A**): gut microbiota and SCFAs, (**B**): gut microbiota and gas production and (**C**): gut microbiota and tryptophan metabolites. Experimental treatments included: NC (negative control, 0 g/L), LRS2 (5 g/L), MRS2 (10 g/L), and HRS2 (15 g/L) of Hi-Maize 260 (*n* = 8 per group, with 4 female and 4 male replicates). One-way ANOVA with Tukey’s post hoc test was performed. Significant correlations were marked by * *p* < 0.05; ** *p* < 0.01; *** *p* < 0.001. Absence of symbols denotes no significant difference among the four groups.

To further elucidate the relationship between SCFAs and the gut microbiota following RS2 treatment, we conducted a Spearman correlation analysis to systematically evaluate the associations between SCFAs and the top 30 most abundant gut microbiota in terms of relative abundance. As could be seen in Figure 6A, the abundance of *Bifidobacterium*, which significantly increased following RS2 intervention, was positively correlated with acetic acid, butyric acid, valeric acid, and isovaleric acid. Similarly, *Lactobacillus* showed positive correlations with acetic acid, butyric acid, and isovaleric acid. Conversely, genera whose abundance was significantly reduced, such as *Parabacteroides* and *Sutterella*, exhibited negative correlations with acetic acid, propionic acid, butyric acid, valeric acid, and isovaleric acid. Additionally, *Lachnoclostridium* was negatively correlated with acetic acid, propionic acid, butyric acid, isobutyric acid, valeric acid, and isovaleric acid.

Links between the 30 highest-abundance gut bacteria and gas output were additionally evaluated. *Bifidobacterium* showed a positive correlation with CO_2_ and CH_4_, while *Lactobacillus* was positively associated with CH_4_. In contrast, *Escherichia-Shigella* exhibited positive correlations with H_2_S and H_2_. Conversely, *Sutterella* and *Lachnoclostridium* were negatively correlated with CH_4_, and similarly, *Parabacteroides* displayed inverse relationships with both CH_4_ and CO_2_.

The correlation between the top 30 most abundant gut microbiota and tryptophan metabolites was also examined. In Figure 6C, *Bifidobacterium* was positively correlated with ILA, Indole, IALD, Tryptophan, and negatively correlated with IAA and KYN. *Lactobacillus* was positively correlated with TA, ILA, Indole, IALD, and 5-HT, and negatively correlated with IAA and KYN. The significant reduction in *Parabacteroides* was associated with positive correlations with ILA, Indole, IALD, Tryptophan, and negative correlations with IAA and KYN. Conversely, the significant reduction in *Parabacteroides* was also negatively correlated with ILA, Indole, IALD, Tryptophan, and 5-HT, and positively correlated with IAA, IPA, KYN. *Sutterella* was negatively correlated with Indole, IALD, and 5-HT, and positively correlated with IAA, KYN. *Lachnoclostridium* was negatively correlated with TA, ILA, Indole, IALD and 5-HT, and positively correlated with IAA, IPA and KYN.

The gut microbiota species *Eubacterium rectale* is a key butyrate producer and an efficient degrader of complex carbohydrates in the colon. Furthermore, key butyrate-producers in the *Eubacterium rectale* group show enhanced activity when exposed to diets containing resistant starch. *Eubacterium rectale* group is a type of bacteria found in the human gut. It is involved in tryptophan synthesis. Additionally, it produces butyrate, which is the main SCFA produced by this species [41].

## 4. Discussion

Resistant starch has been widely investigated for its numerous physiological benefits. Among RS subtypes, RS2, characterized by its natural granular structure with B-type crystallinity, is predominantly found in unprocessed food sources including raw potatoes, green bananas, and high-amylose maize starch [42]. The present study aimed to elucidate the mechanistic basis of RS2′s prebiotic properties through comprehensive analysis of its dose-dependent modulation of microbial composition and metabolic activities; and to establish evidence-based dosage guidelines for optimal nutritional intervention, thereby providing scientific rationale for personalized RS2 supplementation strategies tailored to specific physiological conditions.

Current evidence consistently demonstrates that RS2 exerts its physiological effects through microbiota-dependent mechanisms. Characteristic responses to RS2 intervention include reduced alpha-diversity indices, and selective enrichment of specific bacterial taxa [1,2]. Our study revealed that while alpha-diversity measures did not differ significantly across RS2 dosage groups, PCoA analysis demonstrated marked beta-diversity separation, indicating distinct community structures. Notably, we observed a dose-dependent shift in microbial composition characterized by a progressive decrease in *Proteobacteria* abundance and corresponding increase in *Bacteroidetes* populations. This reciprocal pattern has been mechanistically linked to amelioration of colitis symptoms through improved mucosal barrier function [43], consistent with established knowledge that *Proteobacteria* enrichment often signifies dysbiosis and pathogenic potential while *Bacteroidetes* expansion enhances fiber fermentation capacity. Clinical correlations further support these findings and highlighted that the relative abundance of beneficial genera such as *Bifidobacterium* and *Lactobacillus* is negatively correlated with type 2 diabetes [44], while pathogenic microbiota exhibit distinct disease associations, where *Escherichia-Shigella* mediates intestinal pathogenesis through epithelial invasion and toxin secretion [45]; *Lachnoclostridium* exacerbates metabolic dysfunction via insulin resistance promotion [46]. *Fusobacterium* drives endometriosis progression through lesion proliferation [47]; and *Sutterella* compromises mucosal immunity via IgA protease-mediated degradation [48]. Notably, our dose–response analysis revealed that high-concentration RS2 (15 g/L) fermentation simultaneously enhanced probiotic populations, including *Bifidobacterium* and *Lactobacillus*, while suppressed potential pathogenic taxa, such as *Escherichia-Shigella*, *Lachnoclostridium*, *Fusobacterium*, and *Sutterella*, demonstrating its dual modulatory capacity to promote intestinal eubiosis through selective microbial enrichment and pathogen inhibition, thereby establishing RS2 as a potent dietary modulator of gut ecosystem homeostasis.

Emerging evidence underscores the gut microbiota’s pivotal role in host health through the production of bioactive metabolites, including SCFAs, which maintain intestinal homeostasis and regulate systemic metabolism [49,50,51,52,53]. Our study extends this knowledge by demonstrating a clear dose-dependent relationship between RS2 and SCFA production. Specifically, high-dose RS2 (15 g/L) fermentation elicited the most pronounced increase in total SCFAs, concomitant with the greatest pH reduction. This reflects a dose-responsive enhancement of collective microbial metabolic activity [54,55].

The dynamic changes in pH observed during fermentation are intrinsically linked to the metabolic output of the gut microbiota. The significant decrease in pH in the RS2-treated groups, particularly in HRS2, can be directly attributed to the accumulation of SCFAs [56], which are weak acids that dissociate in the aqueous environment of the medium. This lowered pH is not merely a consequence but also a regulator of microbial ecology. It likely creates a selective pressure, inhibiting pH-sensitive pathogens while favoring acid-tolerant commensals such as *Lactobacillus* and *Bifidobacterium* species [57]. This shift in community structure may explain the enhanced production of specific metabolites like lactate and acetate, which can serve as precursors for other SCFAs. Furthermore, the acidic environment may modulate the enzymatic activity of key bacterial enzymes involved in cross-feeding pathways. For instance, a lower pH is known to favor the activity of enzymes that convert lactate and acetate into the more energetically favorable butyrate [58]. This could mechanistically explain the dose-dependent increase in butyrate production observed in our study, suggesting that RS2 not only provides substrate but also helps to engineer a pH microenvironment that is optimal for the production of this highly beneficial metabolite.

Microbial fermentation generates multiple gaseous metabolites (CO_2_, H_2_, CH_4_, and H_2_S) that significantly influence intestinal physiology and contribute to abdominal symptoms in gastrointestinal disorders [59]. These gases exhibit distinct physiological impacts: H_2_S inhibits GLP-1 secretion and gene expression, compromising metabolic regulation [60]; microbial H2 facilitates secondary metabolic processes [61]; and CH4 reduces intestinal motility, potentially leading to constipation [62]. While H_2_S maintains intestinal homeostasis at physiological concentrations through epithelial barrier preservation, immune modulation, and enteric nervous system regulation via post-translational modifications, KATP channel activation, and colonocyte energetics, excessive production induces epithelial dysfunction and chronic inflammation [63,64]. Moreover, elevated H_2_S levels can disrupt mitochondrial function, impair cellular energy production, and diminish antioxidant capacity, ultimately compromising cellular homeostasis and posing health risks [65]. Consequently, reducing excessive H_2_S generation by the gut microbiota, particularly through dietary strategies such as RS2 supplementation and microbial ecology modulation, may yield multiple benefits for intestinal and systemic health. These include enhanced barrier function, attenuated inflammation, and restored microbial balance. Our findings uncover a novel dose-dependent regulatory effect of RS2 on microbial gas metabolism: a significant reduction in H_2_S production was observed specifically at the 15 g/L dose. This phenomenon appears mediated through two complementary mechanisms: first, the selective enrichment of acetogenic taxa (e.g., *Bifidobacterium*, *Lactobacillus*, *Blautia*) created metabolic competition for gaseous substrates (H_2_/CO_2_), effectively diverting carbon flux toward acetate synthesis rather than sulfidogenesis. Second, functional prediction analysis demonstrated significant suppression of sulfur relay system and sulfur metabolism pathways as visualized in Supplementary Figure S3. This metabolic reprogramming suggested RS2’s unique capacity to simultaneously promote saccharolytic fermentation while inhibiting proteolytic pathways associated with gas-related symptomatology. This dual modulation indicated that RS2 at optimal doses can simultaneously enhance beneficial microbial activities while mitigating gas-related gastrointestinal distress through metabolic pathway redirection.

Tryptophan serves as a critical essential amino acid whose biological significance stems from its indole moiety, functioning as a fundamental precursor for numerous bioactive compounds. While the majority of tryptophan (90–95%) undergoes host-mediated metabolism through the KYN and 5-HT pathways, approximately 4–6% is metabolized by gut microbiota into various indole derivatives, including IAA, IPA, 3-IAlD, ILA, and tryptamine [66]. These microbial metabolites, particularly IPA and ILA, exert significant physiological effects through aryl hydrocarbon receptor activation, thereby enhancing intestinal epithelial barrier integrity and modulating immune cell function [22]. The gut microbiota demonstrates remarkable metabolic plasticity in tryptophan metabolism, with distinct bacterial taxa preferentially directing metabolic flux toward specific pathways. Certain microbial species specialize in KYN production [67], while others, particularly *Lactobacillus* strains, have been shown to modulate the activity of rate-limiting enzymes in tryptophan metabolism, favoring 5-HT biosynthesis over KYN production. This enzymatic regulation may explain our observed increase in 5-HT concomitant with decreased KYN levels, suggesting *Lactobacillus*-mediated metabolic reprogramming [68]. 5-HT serves as a critical neuromodulator of gastrointestinal function, regulating motility, secretion, and vascular tone. Emerging evidence indicates that microbiota-derived 5-HT can directly influence regulatory T cell metabolism and promote immune tolerance [69].

Our results further demonstrate that RS2 fermentation promotes the generation of beneficial indole derivatives through sequential microbial transformations, a pathway strongly corroborated by our correlation analysis (Figure 6C). We propose that this metabolic shift from KYN to indole derivatives may be driven by two complementary mechanisms: (1) reduced substrate flux toward the kynurenine pathway leaves more tryptophan available for microbial indole formation; and (2) RS2-induced enrichment of indole-producing bacteria, such as *Bifidobacterium* and *Lactobacillus,* are known ILA producers, thereby amplifying indole-derivative output [70]. Notably, the RS2-enriched genera *Bifidobacterium* and *Lactobacillus* demonstrated significant positive associations with beneficial metabolites (ILA, indole, IALD, tryptophan, TA, and 5-HT) and negative correlations with detrimental metabolites (IAA and KYN). This metabolic signature, combined with established literature [37], suggests that these taxa preferentially direct tryptophan metabolism toward immunomodulatory and neuroprotective pathways, initiating a metabolic cascade that supports intestinal homeostasis. Conversely, the RS2-mediated suppression of proteolytic pathobionts such as *Lachnoclostridium* and *Sutterella* likely accounts for the reduction in IPA and IAA levels [71]. This conclusion is strengthened by their strong positive correlations with IAA and KYN, and negative correlations with beneficial indoles and 5-HT, suggesting their potential role in promoting gut dysbiosis through perturbation of tryptophan metabolism [72,73]. The concomitant decrease in 2-OX may reflect inhibited Bacteroides activity, thereby reducing cytotoxic metabolites [74]. Collectively, these coordinated microbial ecological shifts, evidenced by both taxonomic changes and robust correlation patterns, create a favorable metabolic profile. The resulting metabolites (e.g., IALD, ILA, indole) collectively enhance epithelial barrier function, mitigate inflammation, and promote immune regulation [37,38,75], thereby supporting gut health and systemic homeostasis through dose-dependent modulation of tryptophan metabolism.

The correlations between gut microbiota and key metabolites (SCFAs, gases, and tryptophan derivatives) highlight their critical roles in maintaining host health. Specifically, HRS2 intervention (15 g/L) induced a pronounced proliferation of beneficial genera, particularly *Bifidobacterium* and *Lactobacillus*, which showed strong positive correlations with butyrate levels. This relationship is physiologically consequential, as butyrate serves as both an energy source for colonocytes and a regulator of tight junction protein expression, thereby enhancing intestinal barrier function [50]. The coordinated increase in these butyrogenic taxa and their metabolic products suggests that RS2 exerts its prebiotic effects through selective stimulation of beneficial microbial metabolic pathways. A striking observation was the dose-dependent reduction in H_2_S production in the HRS2 group, which correlated inversely with *Escherichia-Shigella* abundance. This reduction likely reflected competitive exclusion mechanisms, whereby RS2 fermentation created an ecological niche favoring saccharolytic bacteria over proteolytic, sulfate-reducing pathobionts.

**Figure 7:**
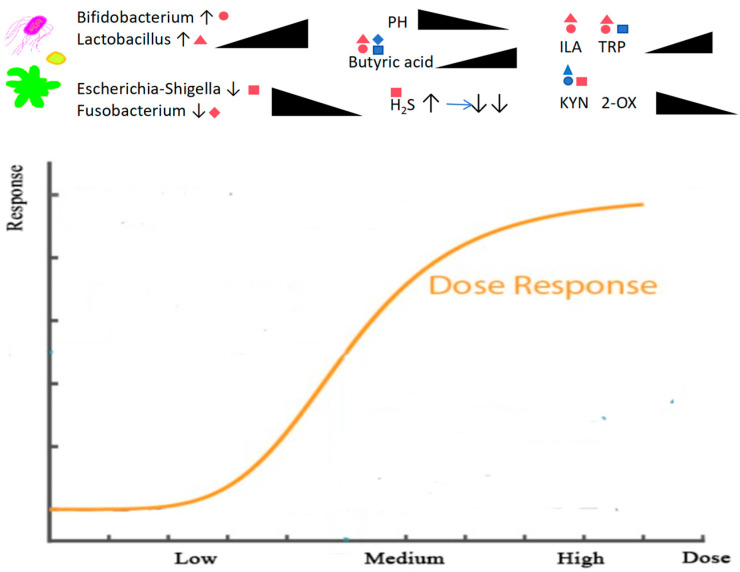
Dose-dependent effects of RS2 on pH, gas, tryptophan metabolites and their correlations with key microbiota. TRP, tryptophan; KYN, kynurenine; 2-OX, 2-oxindole; ILA, indole-3-lactic acid) across different RS2 doses (low, medium, high). Upward arrows indicate a trend of increasing concentration with higher RS2 dose, while shaded shapes indicate a trend of decreasing/increasing concentration. Shape and color identity represents a significant positive correlation (*p* < 0.05). Shape identity with inverse color represents a significant negative correlation (*p* < 0.05). Genera are represented by shapes: circle (*Bifidobacterium*), triangle (*Lactobacillus*), square (*Escherichia-Shigella*), and diamond (*Fusobacterium*). Downward arrows indicate a trend of decreasing concentration with higher RS2 dose. Right-pointing arrows represent a change in trend.

Based on our results, we propose a hypothetical model illustrating the dose-dependent influence of RS2 on the gut microbial ecosystem and beneficial metabolic outcomes (summarized in Figure 7). Increasing RS2 doses led to a progressive decrease in fermentation pH and a marked increase in butyrate production. These changes were accompanied by the enrichment of beneficial bacteria (e.g., *Bifidobacterium* and *Lactobacillus*), which correlated positively with butyrate levels, and the suppression of potential pathobionts (e.g., *Escherichia*—*Shigella* and *Fusobacterium*), which showed negative correlations. Concurrently, H_2_S—a harmful metabolite associated with these pathobionts—exhibited an initial rise followed by a significant reduction at higher RS2 doses. With respect to tryptophan metabolism, high-dose RS2 redirected metabolic flux toward beneficial pathways: levels of beneficial metabolites (e.g., ILA and tryptophan) increased, while detrimental metabolites (e.g., KYN and 2-OX) decreased. This shift aligned with microbial changes, characterized by an increase in beneficial, metabolite-producing bacteria and a decline in pathobionts involved in deleterious pathways

While our findings highlight the pronounced benefits of high-dose RS2 (15 g/L) in vitro, the translational practicality of this dose warrants careful consideration. Based on a modelled human colonic volume of 0.75 L, this concentration equates to a daily intake of approximately 11.25 g of RS2. This level of intake is readily achievable through dietary modifications and is, in fact, lower than the doses often used in clinical supplementation trials (typically 25–30 g/day). However, it is important to acknowledge the inherent limitations of in vitro models, which, while excellent for elucidating mechanistic pathways, cannot fully recapitulate the complexity of human physiology, including host metabolism, immune responses, and transit time. Therefore, our results should be interpreted as a robust proof-of-concept that justifies and informs the design of future human interventions. These clinical studies are necessary to confirm that the promising dose-dependent effects we observed on microbial composition and metabolic output translate into tangible health benefits in humans.

## 5. Conclusions

Our study demonstrated that high-dose RS2 (15 g/L) exerted significant prebiotic effects through multiple mechanisms: enhancing saccharolytic fermentation, as evidenced by increased SCFA production and intestinal acidification; suppressing proteolytic activity, with a significant reduction in H_2_S generation; and modulating tryptophan metabolism toward beneficial pathways. RS2 supplementation induced dose-dependent microbial shifts, promoting beneficial taxa (*Bifidobacterium*, *Prevotella* 9, *Megamonas*, *Lactobacillus*), while inhibiting potential pathobionts (*Escherichia-Shigella*, *Lachnoclostridium*, *Fusobacterium* and *Sutterella*).

## Data Availability

The original contributions presented in this study are included in the article/Supplementary Materials. Further inquiries can be directed to the corresponding authors.

## Supplementary Materials

The following supporting information can be downloaded at https://www.mdpi.com/article/10.3390/foods14183255/s1. Figure S1: Veen analysis of four groups. Figure S2: Effect of Type 2 resistant starch (RS2) on short-chain fatty acid (SCFA) production and pH by human gut microbiota after in vitro fermentation. Figure S3: Comparison of microbial function prediction. Table S1: The demographic characteristics of the volunteers. Table S2: Detailed Gas Chromatography (GC) Parameters for SCFA Analysis.
