# Supplementary material for: The Dose-Dependent Influence of Type 2 Resistant Starch on Gut Microbial Communities and Metabolic Outputs: An In Vitro Simulation
Source: Foods. 2025 Sep 19;14(18):3255. doi: 10.3390/foods14183255 (PMC12469863; doi:10.3390/foods14183255)
Supplement: Supplementary file 1 [file foods-14-03255-s001.zip › foods-3846371-supplementary.pdf]

The Dose-Dependent Influence of Type-2 Resistant Starch on Gut Microbial Communities and Metabolic Outputs: An *In Vitro* Simulation

Huowang Zheng[1 2 #], Fangshu Shi[2], Jinjun Li[2], Xiangyu Bian[2], Shuisheng Wu[1 *], Xiaoqiong Li[2 *]

[1] College of Pharmacy, Fujian University of Traditional Chinese Medicine, Fuzhou 350122, China.

[2] State Key Laboratory for Quality and Safety of Agro-Products & Institute of Food Sciences, Zhejiang Academy of Agricultural Sciences, Hangzhou 310021, China.

*Correspondence: Shuisheng Wu & Xiaoqiong Li

College of Pharmacy, Fujian University of Traditional Chinese Medicine, Fuzhou 350122, China, 1998004@fjtcm.edu.cn

Institute of Food Sciences, Zhejiang Academy of Agricultural Sciences, 0707lianlan@163.com

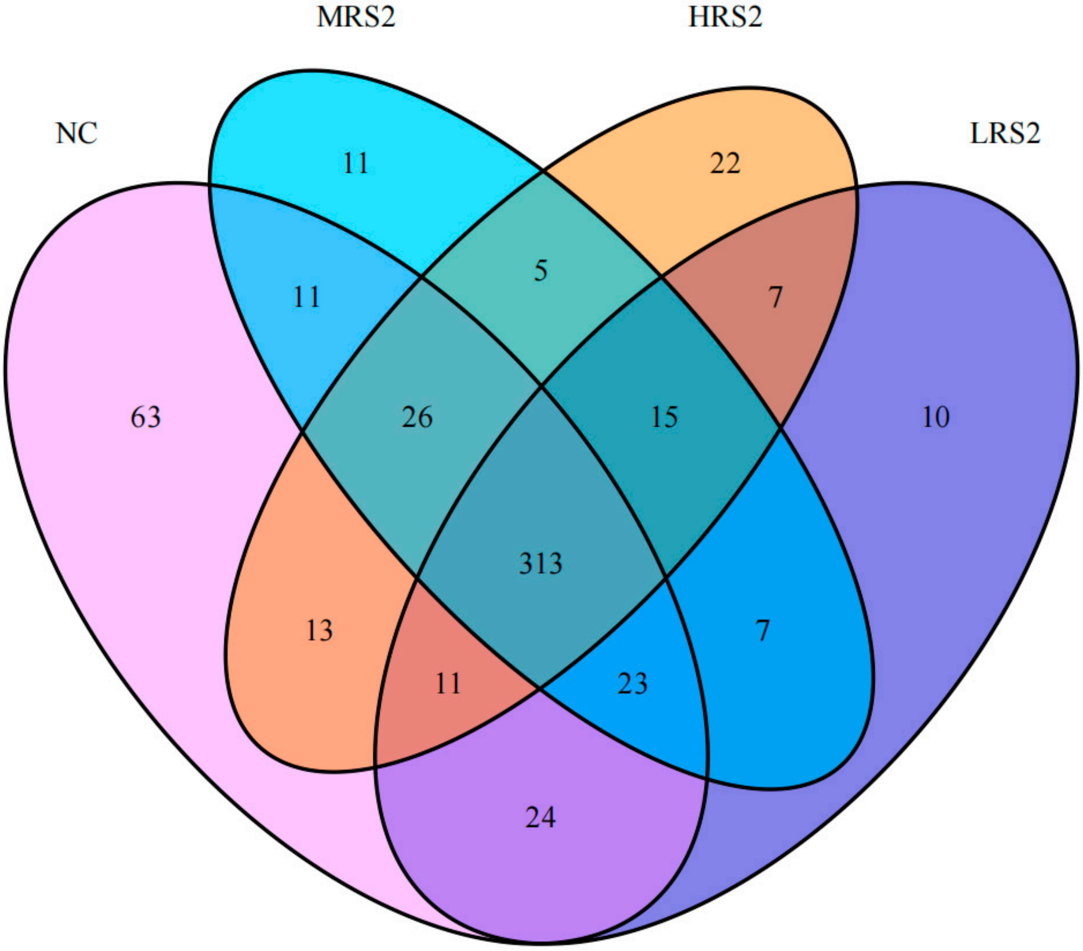

Supplemental Figure S1. Veen analysis of four groups. Experimental treatments included: NC (negative control, 0 g/L), LRS2 (5 g/L), MRS2 (10 g/L), and HRS2 (15 g/L) of Hi-Maize 260 (n = 8 per group, with 4 female and 4 male replicates). Absence of symbols denotes no significant difference among the four groups.een analysis of four groups.

A

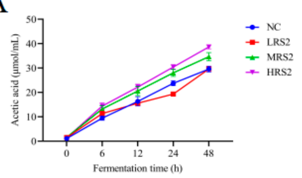

Groups	0 h	6 h	12 h	24 h	48 h
NC	a	b	b	c	b
LRS2	a	a	b	b	b
MRS2	a	a	a	a	a
HRS2	a	a	a	a	a

B

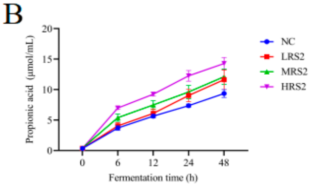

Groups	0 h	6 h	12 h	24 h	48 h
NC	a	b	b	b	b
LRS2	a	b	b	b	ab
MRS2	a	ab	ab	ab	a
HRS2	a	a	a	a	a

C

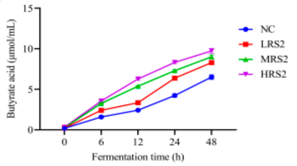

Groups	0 h	6 h	12 h	24 h	48 h
NC	a	c	d	d	d
LRS2	a	b	c	c	c
MRS2	a	a	b	b	b
HRS2	a	a	a	a	a

D

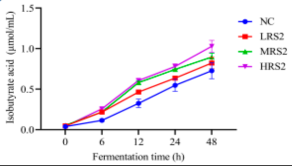

Groups	0 h	6 h	12 h	24 h	48 h
NC	a	a	b	b	b
LRS2	a	a	ab	ab	b
MRS2	a	a	ab	ab	ab
HRS2	a	a	a	a	a

E

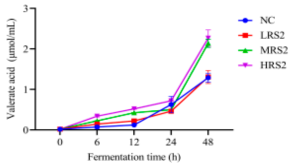

Groups	0 h	6 h	12 h	24 h	48 h
NC	a	a	b	a	b
LRS2	a	a	ab	a	b
MRS2	a	a	ab	a	a
HRS2	a	a	a	a	a

F

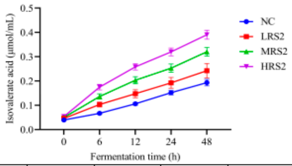

Groups	0 h	6 h	12 h	24 h	48 h
NC	a	a	a	a	a
LRS2	a	b	b	b	b
MRS2	a	c	c	c	c
HRS2	a	c	c	c	c

G

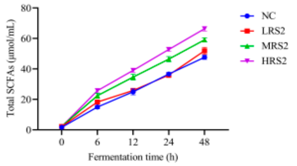

Groups	0 h	6 h	12 h	24 h	48 h
NC	a	b	b	c	c
LRS2	a	b	b	c	c
MRS2	a	ab	a	b	b
HRS2	a	a	a	a	a

H

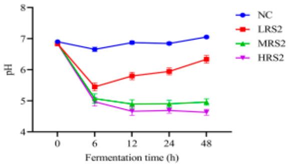

Groups	0 h	6 h	12 h	24 h	48 h
NC	a	a	a	a	a
LRS2	a	b	b	b	b
MRS2	a	c	c	c	c
HRS2	a	c	c	c	c

Supplemental Figure S2. Effect of Type 2 resistant starch (RS2) on short-chain fatty acid (SCFA) production and pH by human gut microbiota after in vitro fermentation.. A: pH, B: acetic acid, C: propionic acid, D: butyric acid, E: isobutyric acid, F: valeric acid, G: isovaleric acid and H: the total SCFAs. Experimental treatments included: NC (negative control, 0 g/L), LRS2 (5 g/L), MRS2 (10 g/L), and HRS2 (15 g/L) of Hi-Maize 260 (n = 8 per group, with 4 female and 4 male replicates). Statistical analysis was performed using one-way analysis of variance (ANOVA) followed by Tukey's post hoc test in GraphPad Prism 9.5 software, and different letters indicating significant differences between groups ($p < 0.05$)

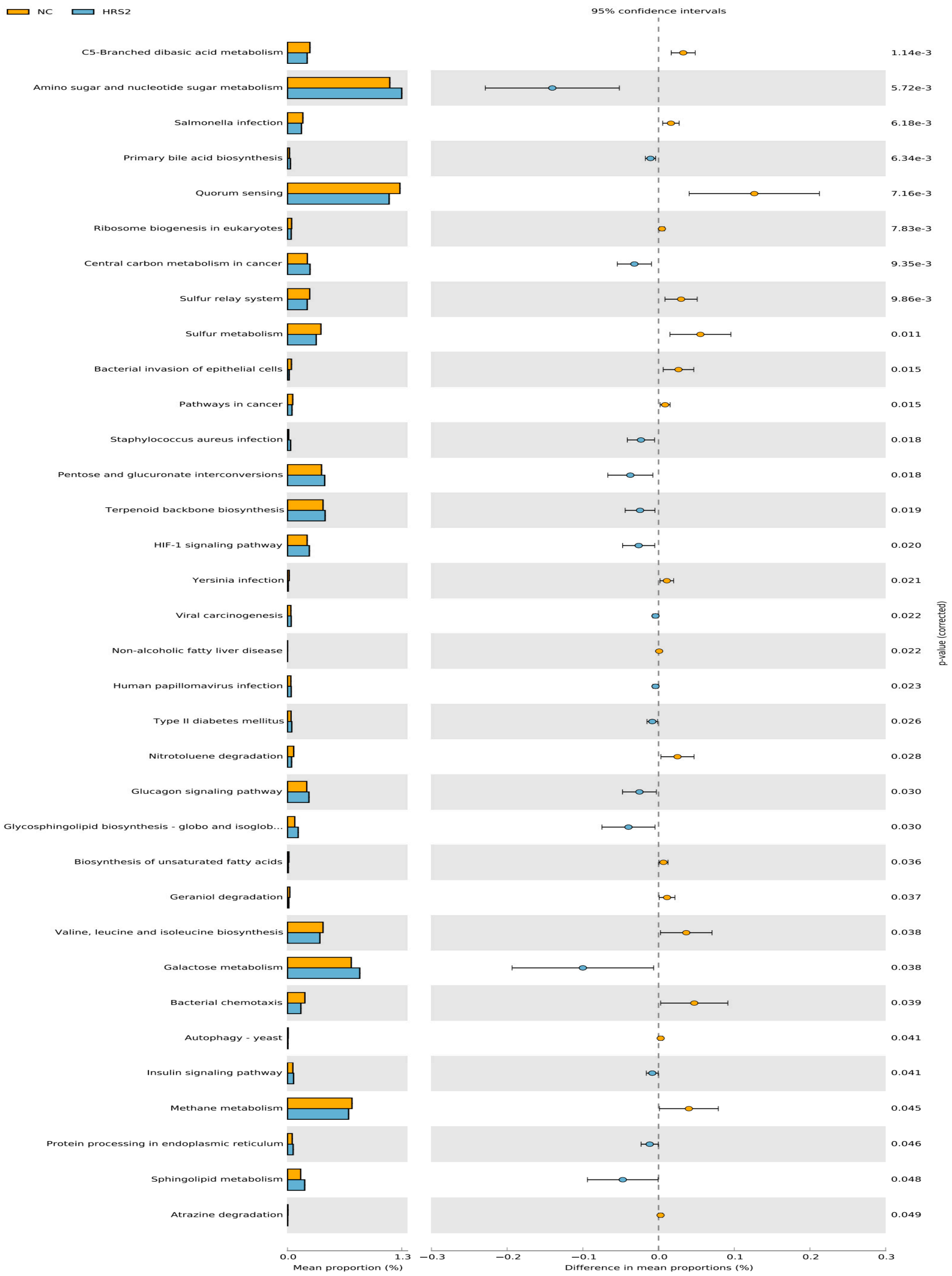

Supplemental Figure S3. Comparison of microbial function prediction. PICRUSt-predicted relative abundances of the KEGG pathway (KEGG level 3). Experimental treatments included: NC (negative control, 0 g/L), LRS2 (5 g/L), MRS2 (10 g/L), and HRS2 (15 g/L) of Hi-Maize 260 (n = 8 per group, with 4 female and 4 male replicates). Statistical analysis was conducted using Welch' s t-test; only results with p values less than 0.05 (statistically significant) are displayed.

Supplementary Table S1.

The demographic characteristics of the volunteers

Characteristic	All (n=8)	Male (n=4)	Female (n=4)
Age (year)	24.88±1.36	24.50±0.58	25.25±1.89
Height (m)	1.65±0.06	1.68±0.05	1.63±0.05
Weight (kg)	57.25±9.21	64.50±6.40	50.00±6.40
BMI(kg/m^2)	20.91±2.46	18.91±1.82	22.91±1.82

BMI, Body mass index (kg/m^2); Values are expressed as means±SD (standard deviation).

Inclusion Criteria:

Aged between 18 and 40 years.

Han Chinese residents in Zhejiang Province without specific dietary habits.

Willing to participate in the study and provide stool samples.

No other intestinal diseases

Exclusion Criteria:

Use of probiotics, prebiotics, or antibiotic medications in the past six months.

Presence of abnormal intestinal function or other side effects.

Supplementary Table S2.

Detailed Gas Chromatography (GC) Parameters for SCFA Analysis.

Parameter	Setting / Value
GC System	GC2010plus (Shimadzu)
Detector	Flame Ionization Detector (FID)
Column	DB-FFAP (30 m × 0.25 mm × 0.25 µm; Agilent)
Carrier Gas	Nitrogen (N_2)
Injection Volume	1 µL
Injector Temperature	240 °C
Detector Temperature	260 °C
Oven Temperature Program	80 °C (hold 1 min) → 180 °C at 10 °C/min → 240 °C at 20 °C/min (hold 5 min)
Gas Flow Rates	2.5 mL/min (hold 6.5 min) → 2.8 mL/min (hold 2 min)
Run Time	~20 min
